# Interaction of Mason-Pfizer monkey virus matrix protein with plasma membrane

**DOI:** 10.3389/fmicb.2013.00423

**Published:** 2014-01-21

**Authors:** Jan Prchal, Tomáš Kroupa, Tomáš Ruml, Richard Hrabal

**Affiliations:** ^1^Laboratory of NMR Spectroscopy, Institute of Chemical Technology, PragueCzech Republic; ^2^Department of Biochemistry and Microbiology, Institute of Chemical Technology, PragueCzech Republic

**Keywords:** Retrovirus, Mason-Pfizer monkey virus, matrix protein, phospholipids, interaction, plasma membrane

## Abstract

Budding is the final step of the late phase of retroviral life cycle. It begins with the interaction of Gag precursor with plasma membrane (PM) through its N-terminal domain, the matrix protein (MA). However, single genera of *Retroviridae* family differ in the way how they interact with PM. While in case of *Lentiviruses* (e.g., human immunodeficiency virus) the structural polyprotein precursor Gag interacts with cellular membrane prior to the assembly, *Betaretroviruses* [Mason-Pfizer monkey virus (M-PMV)] first assemble their virus-like particles (VLPs) in the pericentriolar region of the infected cell and therefore, already assembled particles interact with the membrane. Although both these types of retroviruses use similar mechanism of the interaction of Gag with the membrane, the difference in the site of assembly leads to some differences in the mechanism of the interaction. Here we describe the interaction of M-PMV MA with PM with emphasis on the structural aspects of the interaction with single phospholipids.

In the late phase of retroviral life cycle, all structural proteins are produced in a host cell as a polyprotein precursor Gag. Such pre-arrangement ensures their equimolar incorporation and proper functioning in a viral particle (Hunter, 1994). Gag proteins of most retroviruses (formerly described as C-type retroviruses) are immediately after the synthesis transported to the plasma surface where they interact with the plasma membrane (PM) and assemble to an immature viral particle simultaneously with its budding.

Mason-Pfizer monkey virus (M-PMV) belongs to *Betaretrovirus* genus of *Orthoretroviridae* subfamily and is also reffered to as SRV type 3(Montiel, 2010). It is a simple simian exogenous, non-transforming, horizontally transferred retrovirus, which causes failure of the immune system of the infected animal. Initially it was isolated from breast tumor of rhesus monkey (*Macaca mulatta*), but as it was learned shortly after its discovery, it is not the direct cause of a carcinoma development (Chopra and Mason, 1970). Although it causes similar disease as simian immunodeficiency virus (SIV), it is not related to it and belongs to different genus. M-PMV was formerly described as B/D-type retrovirus, which means that its Gag proteins are first transported to a periplasmic region of the cell, where they assemble (Vlach et al., 2008). Resulting immature virus particle is then transported to the PM where budding occurs. The membrane interaction of Gag proteins, as well as intracellular transport is facilitated by their N-terminal domain – the matrix protein (MA). MA is localized on the surface of the virus particle and remains associated with the virus membrane after maturation of Gag, which is cleaved by viral protease to individual structural proteins. MAs of most retroviruses are N-terminally myristoylated.

The interaction of MAs with the PM is enabled by a bipartite signal which consists of the myristoyl and a surface displayed patch of basic residues, mostly arginines and lysines. This is a canonical arrangement of the binding epitope shared by most retroviral proteins interacting with phospholipids (Bryant and Ratner, 1990; Zhou et al., 1994; Freed, 1998). While positively charged amino acids of MA interact with phosphate groups of the membrane, the myristic acid is in a close contact with phospholipid long aliphatic chains. Both interactions are additive, i.e., they contribute to the overall affinity of MA toward the PM. The interaction of the myristoyl with the membrane is not strong enough to mediate the membrane binding of MA (Gag) without contribution of other forces (Peitzsch and Mclaughlin, 1993). The basic residues on the surfaces of lipid binding proteins warrant this function as they are responsible for non-specific electrostatic interactions with negatively charged polar heads of phospholipids. However, differently phosphorylated phosphoinositides are present in membranes of various cellular organelles serving as specific markers which are recognized by numerous cargo transferring proteins (Roth, 2004).

Myristoylated human immunodeficiency virus (HIV-1) MA interacts with the membrane by using a mechanism called myristoyl switch (Zhou and Resh, 1996). In cytosol or *in vitro*, the myristoyl of MA is sequestered inside the protein core. However, it is released and serves as one of the interaction epitopes of Gag (virions) upon approaching the membrane of infected cell. The process must be carefully controlled to ensure both the binding of MA to the membrane to enable budding, however, loose enough to allow release of the mature virus from its membrane during the early phase of infection.

In retroviruses, the mechanism was well described for the interaction of HIV-1 MA with PM (Zhou and Resh, 1996; Tang et al., 2004; Saad et al., 2006). Saad reported that the switch was triggered by the interaction with phosphatidylinositol-4,5-bisphosphate [PI(4,5)P_2_], a phospholipid present exclusively in the PM. PI(4,5)P_2_ binds to a binding site on the surface of MA and causes myristoyl to be released from the protein and ready for binding. The interaction of HIV-1 MA with PI(4,5)P_2_ composed of shorter fatty-acid chains (4 and 8 carbons in length), was experimentally proved as suitable for solution nuclear magnetic resonance (NMR) measurements because these soluble PI(4,5)P_2_ bind in a cleft between the second and fifth helix. The binding has also been confirmed for phosphatidylinositolphosphates (PIP) containing natural fatty-acid residues (C_18_ and C_20_) either by interaction of MA with artificial liposomes mimicking PM or by blocking PI(4,5)P_2_ synthesis leading to the HIV-1 virus particles to be unable to assembly on PM (Chukkapalli et al., 2008). The interaction of PIP was also proved for other retroviruses: HIV-2, moloney murine leukemia virus (MoMuLV) and equine infectious anemia virus (EIAV). HIV-2 MA interacts with PIP in a similar way as HIV-1 MA, but it was reported that the interaction with neither C_4_ nor C_8_ PI(4,5)P_2_ leads to the release of the myristate (Saad et al., 2008). The authors concluded that the reason was a weaker affinity of PI(4,5)P_2_ to the HIV-2 MA and further speculated that the rationale behind this phenomenon might be that HIV-2 is less infectious than HIV-1. Both HIV-1 and HIV-2 show stronger preference for PI(4,5)P_2_ compared to the other, differently phosphorylated PIPs. EIAV MA is naturally non-myristoylated, so its interaction is fully dependent on the interaction of basic amino-acid residues with membrane phospholipids (Chen et al., 2008). Chen has reported that PI(4,5)P_2_ specifically interacts with EIAV MA and also induces its oligomerization, which promotes the assembly of virus particle. MoMuLV MA also interacts with PIPs, but without any discrimination of PI(4,5)P_2_. However, in the presence of phosphatidylserine, it exhibits stronger and more specific interaction over other differently phosphorylated PIPs (Hamard-Peron et al., 2010). Similar behavior, i.e., preferential and stronger binding of a chosen phosphoinositide in the presence of other phospholipids, mostly in the form of micelles was also described for proteins bearing pleckstrin homology domain (Sugiki et al., 2012). An important role of different phospholipids for the interaction of HIV-1 MA with the PM has been proposed recently by Vlach and Saad (2013). They found that phosphatidylserine, phosphatidylcholine, and phosphatidylethanolamine bound to HIV-1 MA, however, to a different binding site than PI(4,5)P_2_ and that the interaction was weaker. The authors concluded that this interaction further stabilizes the binding of MA to the membrane.

The first evidence of the interaction of M-PMV MA with PI(4,5)P_2_ was reported by Stansell et al. (2007). She observed that depletion of PI(4,5)P_2_ from PM by overexpression of active form of PI-5-phosphatase IV led to 90% decrease of particles release from M-PMV infected cells. Direct evidence of the interaction of M-PMV MA with PI(4,5)P_2_ was then confirmed by Prchal et al. (2012).

Similarly to HIV-1 and HIV-2 MAs, the interaction of M-PMV MA with PI(4,5)P_2_ was studied using NMR spectroscopy and soluble forms of PI(4,5)P_2_ with 4 and 8 carbon fatty-acids. While dibutanoyl PI(4,5)P_2_ did not interact, dioctanoyl PI(4,5)P_2_ interacted specifically with K_D_ of about 100 μM, which is a comparable affinity as that of the interaction of HIV-1 MA with C_4_-PI(4,5)P_2_ (Saad et al., 2006). Similarly as for HIV-2, the interaction did not trigger the myristoyl switch.

The M-PMV MA molecule contains one PIP binding site located between the first, second and fourth helices (**Figure 1**). Comparison of the structures of the myristoylated and non-myristoylated M-PMV MAs showed that this binding site is present only on the surface of the myristoylated protein. Due to a slightly different orientation of the helices in the structure of the non-myristoylated MA, the proper binding pocket is inaccessible for PIP.

**FIGURE 1:**
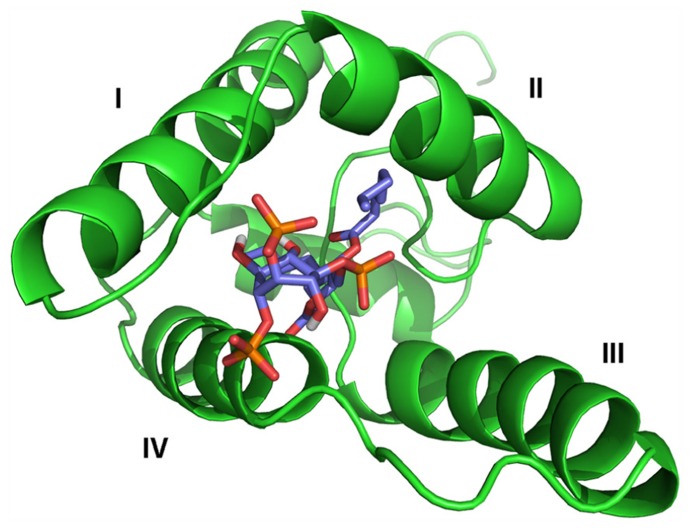
** Structural model of the myristoylated MA – C_8_-PI(4,5)P_2_ complex**. MA is shown in green, with helices numbered by roman numbers. C_8_-PI(4,5)P_2_ molecule colors correspond to its composition – carbons are shown in blue, oxygens in red and phosphors in orange.

The PIP binding site follows the canonical shape of epitopes for binding phosphoinositides, i.e., it is composed of a hydrophobic pocket formed by all four helices and a patch of basic residues on the surface (Roth, 2004). This pocket is connected with the cavity where the myristoyl is sequestered. One of PIP’s fatty-acid chains is buried inside this hydrophobic pocket while the phosphates interact with positively charged amino acids forming the basic patch. The structure has been solved only for the complex of MA with C_8_-PIP, so it might be expected that one of the naturally long PIP’s fatty-acid chains will somehow interfere with the myristoyl which might lead to its exposure from the cavity. The PI(4,5)P_2_ molecule is sequestered deeper in the protein core, compared to HIV-1 MA, where PI(4,5)P_2_ remains on the surface of MA (**Figure 2**).The surface part of the interaction site is formed mainly by lysines and arginines from the loop between the first and second helices and terminal parts of the first, second and fourth helices. The electrostatic interaction between positively charged lysine residues (K16, K25, K27, K33, and K74) and negatively charged inositol phosphate groups is important for the interaction of M-PMV MA with the membrane, as it was proven by mutation studies (Stansell et al., 2007). Stansell found that mutations of basic residues in the proximity of PIP binding site influenced both the transport of immature viral particles and their binding to PM. Virus-like particles (VLPs) bearing mutations K16A or K20A budded into intracellular vesicles. This may indicate that the mutations disrupted the recognition of the target membrane, likely by changing the affinity of MA for differently phosphorylated PIPs than PI(4,5)P_2_. VLPs bearing R10A, R22A, K27A, K33A, or K39A mutations were accumulated near the PM, indicating that the mutations prevented the interaction of MA with PI(4,5)P_2_, or other phospholipids in the membrane. The mutation K25A disrupted some early stages of VLP transport, since they were randomly distributed in the cytoplasm.

**FIGURE 2:**
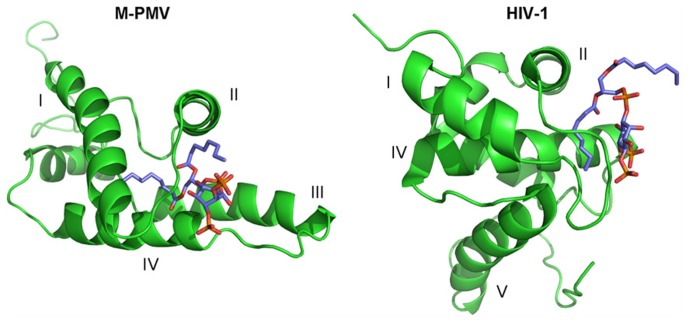
** The comparison of the myristoylated M-PMV MA – C_8_-PI(4,5)P_2_and HIV-1 MA C_4_-PI(4,5)P_2_ complex structures**. MAs are shown in green, with helices numbered by roman numbers. C_8_-PI(4,5)P_2_ molecule colors corresponds to their composition – carbons are shown in blue, oxygens in red and phosphors in orange.

Accumulation of VLPs near PM can also be caused by mutations of non-basic amino-acid residues in M-PMV MA. Double mutations T41I/T78I, Y11F/Y28F, and Y28F/Y67F blocked the release of VLPs from the host cell, while single mutations only slowed down the release of VLPs, but failed to fully arrest it (except of T41I mutation, that showed wt-like virus release; Rhee and Hunter, 1991; Stansell et al., 2004). Since all these mutations introduce more hydrophobic amino acids, Stansell speculated that they created a pocket capable of stronger hydrophobic interactions of mutated residues with the myristoyl and thus block its release from the protein core and therefore, prevents the interaction with PM. However, our results based on the known structure of the complex between myristoylated MA and PI(4,5)P_2_ show that all the mutated residues are too distant from the myristoyl to interact with it (except for T41) but they are part of the PIP binding site (Prchal et al., 2012). Therefore, it is more likely that the mutations rather prevent the interaction of MA with (membrane) phospholipids, than block the myristoyl switch due to a stronger hydrophobic interaction of the myristoyl with exchanged amino acids.

In summary, the MA interaction with the PM is an essential step of retroviral life cycle that allows virus release. A firm contact of Gag with the PM is mediated by the bipartite signal, where the key player is the interaction of MA with PI(4,5)P_2_. This ensures the selectivity for the PM over the membranes of cellular organelles.

## Conflict of Interest Statement

The authors declare that the research was conducted in the absence of any commercial or financial relationships that could be construed as a potential conflict of interest.
